# Supplementary material for: Adherence Measures for Patients with Metastatic Castration-Resistant Prostate Cancer Treated with Abiraterone Acetate plus Prednisone: Results of a Prospective, Cluster-Randomized Trial
Source: Cancers (Basel). 2020 Sep 8;12(9):2550. doi: 10.3390/cancers12092550 (PMC7564106; doi:10.3390/cancers12092550)
Supplement: Supplementary file 1 [file cancers-12-02550-s001.zip › cancers-893202-supple/cancers-893202-supple-figure S1.pdf]

Supplementary Materials: Adherence Measures for Patients with Metastatic Castration-Resistant Prostate Cancer Treated with Abiraterone Acetate Plus Prednisone: Results of a Prospective, Cluster-Randomized Trial

Henrik Suttmann, Jochen Gleissner, Andreas Huebner, Tim Mathes, Werner Baurecht, Katrin Krützfeldt, Hussein Sweiti and Susan Feyerabend

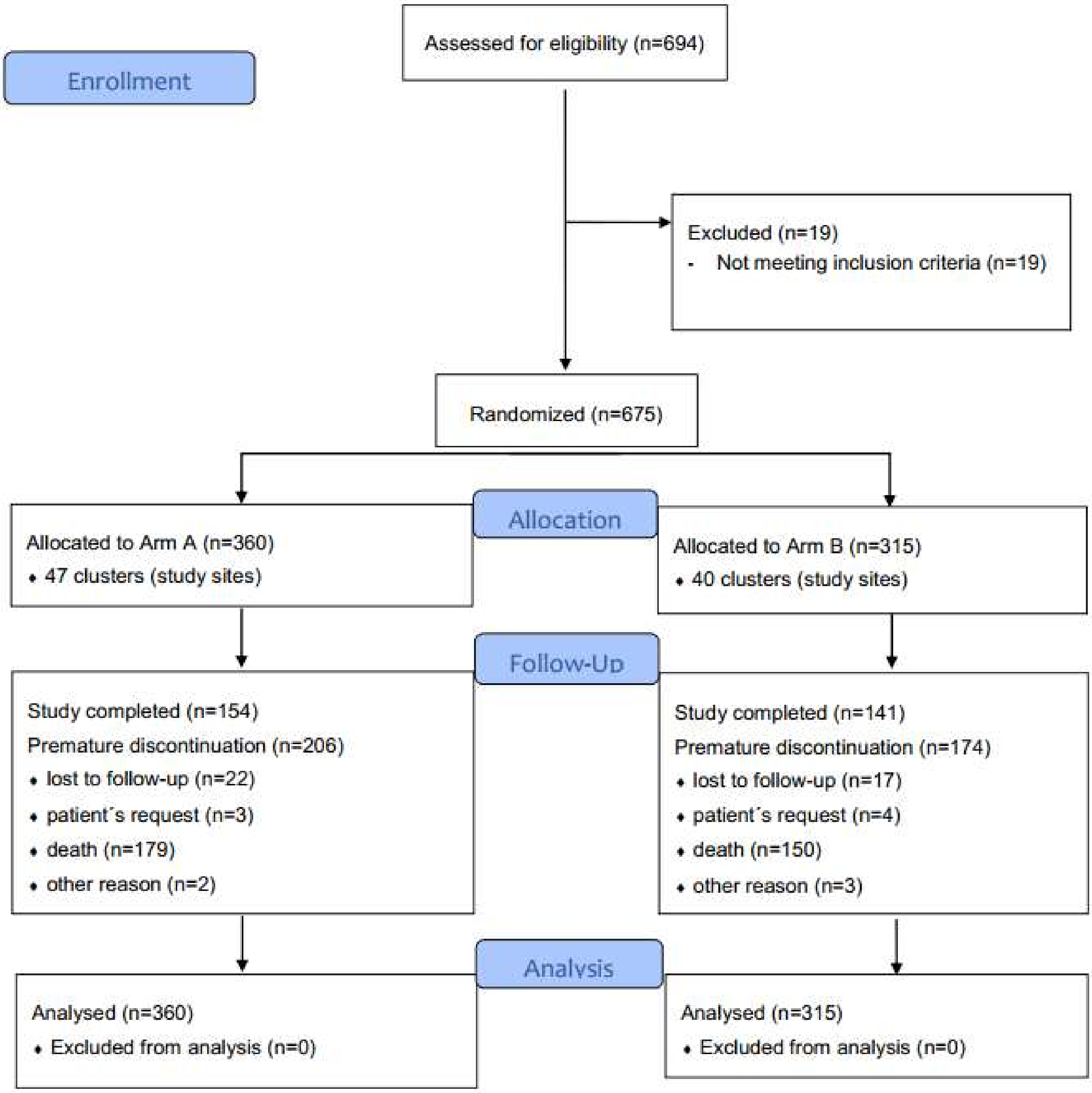

Figure S1. Patient disposition. Patients were allocated to Arm A (adherence-enhancing measures comprising educational/counseling measures as well as reminder elements) or Arm B (no adherence-enhancing measures) by center-based cluster-randomization.

© 2020 by the authors. Licensee MDPI, Basel, Switzerland. This article is an open access article distributed under the terms and conditions of the Creative Commons Attribution (CC BY) license (http://creativecommons.org/licenses/by/4.0/).
