# Supplementary material for: Adherence Measures for Patients with Metastatic Castration-Resistant Prostate Cancer Treated with Abiraterone Acetate plus Prednisone: Results of a Prospective, Cluster-Randomized Trial
Source: Cancers (Basel). 2020 Sep 8;12(9):2550. doi: 10.3390/cancers12092550 (PMC7564106; doi:10.3390/cancers12092550)
Supplement: Supplementary file 1 [file cancers-12-02550-s001.zip › cancers-893202-supple/cancers-893202-supplementary material S1 Study protocol Version 4.0.pdf]

**CLINICAL RESEARCH AND DEVELOPMENT**

# Study plan incl. Amendment II; Non-Interventional Study (NIS)

| | | | |
|---|---|---|---|
| **Version dated:** | 02 July 2015,<br>Version 4.0 | **Medicinal product:** | Abiraterone |
| **NIS No.:** | 212082PCR4002;<br>(IMPACT) | **Document number:** | EDMS-ERI-50121490 |

| | |
|---|---|
| **Title:** | Prospective, non-interventional study on the influence of adherence measures on the therapy with Abiraterone with patients with metastatic, castration resistant prostate carcinoma (mCRPC) |
| **Summary:** | Abiraterone is an orally available active substance, which intervenes in the androgen biosynthesis and which has been used since 2011 for the treatment of advanced prostate carcinoma. The objective of this NIS is to determine the influence of adherence measures on the therapy with Abiraterone in patients with metastatic, castration resistant prostate carcinoma in comparison to a group without adherence program. The primary endpoint is the rate of therapy discontinuations after 3 months for other reasons than disease progression or the start of a new therapy. A further objective is the recording of the quality-of-life, especially the fatigue status of the patients under therapy with Abiraterone. The study merely has an observational character in relation to the pharmacological therapy. Abiraterone is thereby exclusively used within the scope of the marketing authorisation. Suitable patients are adult men with metastatic, castration resistant prostate carcinoma, whose disease is progressive under or after chemotherapy containing Docetaxel, respectively whose disease progression is asymptomatic or mildly symptomatic after the failure of the androgen deprivation therapy, and for whom a chemotherapy is not yet clinically indicated and who are not participating in other clinical studies. The patients are to be representatively recruited at approximately 100 urological and oncological centres throughout Germany (practices as well as clinics with approximately 50 centres per arm and centre-wise, i.e. cluster randomised assignment). |
| **Head of study:** | PD Dr. med. Henrik Suttmann<br>Urology Hamburg<br>Harksheider Str. 3<br>22399 Hamburg |
| **Sponsor:** | JANSSEN-CILAG GmbH<br>Johnson & Johnson Platz 1<br>41470 Neuss |
| **Coordination:** | Dr. H.-W. Grosch<br>JANSSEN-CILAG GmbH<br>Address as above<br>Phone: 02137/955-489; Fax: 02137/955-488 |
| **Data Management and biometrics:** | Acromion GmbH<br>Europaallee 27-29<br>50226 Frechen<br>Phone: 02234/203737-0 Fax: 02234/203737-9 |

This study plan contains confidential information that may only be made accessible to the individuals who are responsible for the execution and organisation of the trial under the maintenance of absolute confidentiality.

# Table of Contents

1 Amendment 2 dated July 2, 2015 .....3
2 Preamble .....3
3 Introduction, background and objectives .....5
4 Medicinal product information .....8
5 Study design .....9
6 Patient collective and documentation criteria .....9
7 Study parameters and study duration .....10
8 Flowchart .....13
9 General information on the drug safety .....14
9.1 Definitions ..... 14
9.2 Documentation and notification of adverse events ..... 17
9.3 Documentation and reporting of adverse reactions during the follow-up observation phase ..... 18
9.4 Other documentation requirements ..... 18
10 Supervision/procedure .....18
11 Quality assurance .....19
11.1 Documentation ..... 19
11.2 Measures on quality assurance, monitoring ..... 19
11.3 Audit ..... 19
12 Statistics .....20
12.1 Random sample calculation ..... 20
12.2 Statistical analysis ..... 21
13 Ethics Committee .....22
14 Data protection .....22
15 Results/publication .....23
16 Administrative issues/notification obligation .....23
17 Archiving .....24
18 Responsibilities .....25
19 Literature list .....26
20 Signatures .....28

# 1 Amendment 2 dated July 02, 2015

**Rationale for the amendment:**

Within the scope of the first interim analysis from March 2015 with a total of 203 patients, for the primary endpoint "Rate of therapy discontinuations after 3 months for other reasons than disease progression or start of a new therapy" no difference was shown in the two observation groups (6.0% (6 /100 patients) in the adherence arm vs. 3.9% (4/103 patients) in the non-adherence arm). The total rate of therapy discontinuations after 3 months was 19.0% (19/100 patients) vs. 12.6% (13/103 patients). The analysis of the patient distribution regarding the pre and post chemo segment showed a clear imbalance in favour of a pre-chemo distribution (73.4% (149/203 patients) vs. 26.6% (54/203 patients) post-chemo patients. The assumptions used for the statistical justification of the case number were not able to be confirmed. At the time of the first interim analysis it remains unclear whether the results of the interim analysis are purely random or are due to unequally distributed influence factors in the two study arms or at individual study centres.
To verify whether the discontinuation rates per study arm and for the subgroups of the pre and post chemo patients, observed during the first interim analysis are able to be confirmed over the further progression of the study, it was therefore decided to continue with the study.

Due to the continuously increasing life expectancy of the patients through modern therapy options, the quality-of-life of tumour patients during their therapy is becoming increasingly important for the concerned patients, as well as for the healthcare institutions that are involved in the marketing authorisation and reimbursement issues for these therapeutic agents. The fatigue/exhaustion accompanying the disease and therapy is experienced as being especially burdensome. This is reported by prostate carcinoma patients as being amongst the most frequent accompanying symptoms, which has a strongly negative influence on the everyday activities and quality-of-life of the patients [26]. In the registration trial COU-AA-301 Abirateronee showed a significant and clinically meaningful improvement versus Placebo of fatigue symptoms reported by patients. As corresponding data about the health-related quality-of-life of affected patients has primarily been available from randomised clinical studies until now, and their transferability to the routine care situation is only possible with limitations, within the scope of the NIS presented here additional quality-of-life data is to be surveyed. For this purpose the inclusion of an additional questionnaire on fatigue (Brief Fatigue Inventory [BFI]) is planned, the most cumbersome and frequent symptom of tumour patients.

As the SMS reminder service as adherence measure during the study has as not yet been used by patients, this service will be discontinued.
The section 9 on drug safety was revised due to updated guidelines from JANSSEN-CILAG.

# 2 Preamble

Non-interventional studies (NIS), amongst which in Germany especially application observations (AWB) are counted, have the objective of gaining

understanding about the application of medicinal products that are already authorised for marketing.

Already with the publishing of the "Recommendations on the planning and execution of application observations" through the BfArM in the Federal Gazette in 1998 and the draft of the joint recommendation of the BfArM and PEI dated May 09, 2007, conditions for the planning, execution and assessment were defined, the observance of which was intended to ensure the validity and scientific acceptance of such surveys. Such investigations, also designated as non-interventional studies [1,2,3], are in their structure and execution oriented towards epidemiological cohort studies. The basis thereby is always the non-intervention through the sponsor [1,2]. The treating physician therefore, decides without specific guidelines whether and with which medicinal products the treatment is to be undertaken, and how the modalities of the treatment (dosage, application form etc.) and the changes of them are managed. Furthermore, the indication specifications must be consistent with the authorised indications and the supply of the medication to the patient is performed with market goods through prescription or issue through the hospital pharmacy. Further requirement is that a procedure is followed according to a study plan, which is consistent with the current status of medical and biometric understanding, and which specifies a structured and systematic observation.

In the current BfArM recommendations [1], as well as in the "VFA recommendations on the improvement of the quality and transparency of non-interventional studies" [3] further measures beyond the existing statutory specifications on the planning, execution and assessment of non-interventional studies (NIS) are defined. Counted amongst them the consultation through an ethics committee formed according to state law and the implementation of measures on quality assurance, which secure the validity and representativeness of the surveyed data. Furthermore, before the inclusion of patients in a NIS it is recommended to acquire a written patient information/declaration of consent, above all regarding the handling of personal data.

With interventional studies with specified examination and treatment measures and the resulting, in part very extensive patient information and acquisition of consent, a selection bias regarding the representativeness of the investigated patient collective is not always able to be excluded. In comparison to this, due to the lacking restricting and modifying mechanisms, an NIS reflects the routine application of marketable medicinal products through the physician and patient. It thereby represents a suitable instrument to generate representative data on the designated use of the therapy regime of an authorised medicinal product under everyday conditions.

# 3 Introduction, background and objectives

In this non-interventional study Abiraterone (Zytiga®) is to be prospectively investigated regarding the influence of adherence measures and the non-adherence rate under real life conditions on the therapy with patients with metastatic, castration resistant prostate carcinoma (mCRPC). In addition, quality-of-life data on the fatigue status of patients is to be surveyed who are being treated with Abiraterone.

Clinical background and results of the marketing authorisation studies with Abiraterone

In spite of further improved treatment regimes metastatic, castration resistant prostate carcinoma (mCRPC) still has a poor prognosis, whereby a residual androgen production has been identified as an important cause for the further tumour progression with mCRPC. Abiraterone is a new type CYP-17-Inhibitor, which not only blocks the androgen production in the testicles, but also in the adrenal glands, as well as in the tumour itself. Until now for the treatment of castration resistant prostate carcinoma, cytotoxic therapeutic agents from the class of the taxane agents (Docetaxel, Cabazitaxel), anti tumour antibiotics (Mitoxantron) and alkylants (Estramustine) have been authorised [4]. Abiraterone shows significant improvements in the overall survival with patients with progressive mCRPC during or after chemotherapy with Docetaxel (extension by 4.6 months) and with patients with asymptomatic and mildly symptomatic progression of the disease after failure of the androgen deprivation therapy, with whom a chemotherapy is not yet clinically indicated (extension of the overall survival by 5.2 months), i.e. with patients with very limited alternative treatment options [5,6]. Especially under consideration of the palliative therapy situation it is of importance for the patients, that Abiraterone only showed a few and primarily light to medium degree medication associated adverse events, which in most cases were also easy to treat [7]. Under the treatment with Abiraterone an improved, faster and sustained pain relief, a significant reduction of the risk for skeletal events such as pathological fractures and spinal-cord compression, as well as a significant improvement of tiredness and exhaustion (fatigue) was shown. Overall improvements in the clinically significant endpoints are mirrored in the improved patient self assessment, such as e.g. in the prostate carcinoma specific functional assessment of the carcinoma therapy (FACT-P).

In contrast to the secured high efficacy of Abiraterone in clinical studies, the effect under real life conditions has not yet been investigated. This above all includes especially the area of the health-related quality-of-life of patients in the routine medical care. To be taken into account thereby, is that due to predefined inclusion and exclusion criteria the evidence from randomised studies does not necessarily mirror the conditions in routine medical care. Therefore the representativity of the results from quality of life questionnaires out of clinical studies for the routine care of patients can be considered as questionable.

Term ‘Therapy adherence‘

In contrast to mostly intravenous chemotherapies, Abiraterone is administered orally. Hereby it is more difficult for the treating physician to exactly monitor the compliance, respectively the adherence to the therapy. For the patients there are, however, advantages as they require less time for the contact with nursing staff or physicians, and do not need to undergo any invasive therapy measures.

The phenomenon of the lacking therapy compliance with chronic diseases, also designated as adherence, was already investigated by the WHO in 2003 and was defined as follows "*The extent to which a person's behaviour – taking medication, following a diet, and/or executing lifestyle changes, corresponds with agreed recommendations from a health care provider*" [8]. Therefore, this adherence definition from the WHO clearly specifies the role of the patient as partner in the therapy, but also the responsibility of the physician for the cooperation of the patient in the adherence to the therapy.

The reasons for an insufficient adherence are diverse. According to more recent studies it is above all influenced through individual opinions and personal prejudices towards the therapy [9].

| Degree of the cooperation | Examples for factors that can influence the adherence |
|---|---|
| Unintentional lacking cooperation | • Problems on the patient's part in understanding the disease/therapy<br>• Wrong expectations towards the therapy<br>• Poor physician/patient relationship with misunderstandings<br>• Incorrect application<br>• Uncomfortable handling<br>• Language problems<br>• Lacking support of the social environment<br>• Psychological problems, above all depression<br>• …. |
| Intentional lacking cooperation | • Lacking cooperation with having to regularly take medication<br>• Lacking feeling of unwellness<br>• Stigmatisation<br>• Fear of side-effects<br>• Fear of habituation or dependency<br>• No tangible or immediate effect<br>• Forgetfulness/negligence<br>• Lacking motivation - especially during asymptomatic phases<br>• Financial reasons<br>• … |

Table modified according to Gillisen A et al., Therapy adherence with Asthma bronchiale and according to Leuppi et al., Adherence in asthma therapy supplemented with information from Osterberg, L et al., Adherence to medication

Modern oncology is currently experiencing a movement away from the intravenous therapies towards oral therapy that is easy to ingest [10]. It is assumed that as of 2013 the proportion of the oral cancer therapies will already be 25% [11]. This requires new concepts regarding the therapy adherence to achieve the maximum advantages of a therapy for the individual patient and the healthcare system [12].

As already applies for other oral cancer therapies such as Tamoxifen [13] or Imatinib [14], the therapy adherence continuously declines over the treatment duration, although its significance for a long term success is well-known with the patients, as well as with the therapists. The measured adherence rates with studies on oral cancer therapy were between 16-100% depending on the therapy and method [15]. In addition to this it was found that an improved patient briefing, education measures and training, inclusion of the family as well as reminder aids can improve the adherence, respectively therapy compliance of the patients [10].

The therapy with Abiraterone has a complex dosage scheme with 4 tablets on an empty stomach, plus 10 mg Prednisone or Prednisolone daily at a meal time.

This can be especially problematic for older carcinoma patients outside of the close care within a clinical study.

Therefore, in cooperation with the Institut für operative Medizin (IFOM, Institute for operative medicine) JANSSEN has developed an adherence program that is to be investigated in the NIS presented here, so as to thereby decrease the assumed gap between effect and efficacy [16].

Fatigue

The improvement of the health-related quality-of-life for patients with incurable diseases is, apart from the longest possible survival time, the most important therapy result. The tumour associated fatigue hereby not only represents the most frequently anticipated and reported symptom of tumour patients (amongst them also prostate carcinoma patients) [25], but it is rather also one of the most encumbering problems for the affected patient during, as well as after the therapy, as the fatigue symptoms can also continue to persist over months and years after the end of the therapy [26]. In fact the tumour associated fatigue can have a greater negative influence on the everyday life and the quality-of-life of concerned patients than all the further disease associated symptoms, such as pain, depression and also nausea [27, 28, 29]. In addition, e.g. through a negative influencing of the patient adherence or as dosage limiting side-effect, the fatigue is able to negatively influence the therapy. Therefore, with increasing life expectancy of the tumour patients this problem is becoming increasingly important in the life and the tumour therapy of concerned patients.

In comparison to a placebo therapy regime, in the marketing authorisation study COU-AA-301 Abiraterone was able to show a significant and clinically meaningful improvement of the patient reported fatigue problems [30]. During the time of therapy in comparison to the placebo arm, Abiraterone was able to significantly reduce the fatigue intensity experienced by the patients at the beginning of the study (defined as decrease of the baseline fatigue value in "Brief Fatigue Inventory" (BFI) (≥ 5 points) by ≥ 2 points in at least two consecutive surveys) (58.1% vs. 40.3%, p=0.0001), as well as also achieve this improvement in a significantly shorter time than the placebo medication (59 days vs. 194 days; P=0.0117; HR 1.392 (1.065-1.818). In the Abiraterone arm, also a significant improvement of the fatigue interference was able to be achieved (defined as decrease of the item "Fatigue Interference" in the BFI from the starting value (≥ 5 points) by ≥ 1.25 points in at least two consecutive surveys) in comparison to the placebo arm (55% vs. 38%, p=0.0075). In addition to this, Abiraterone significantly delayed the time to progression of the fatigue intensity (defined as the time until increase of the item "strongest fatigue in the last 24 hours" in the Brief Fatigue Inventory by ≥ 2 points from the starting value in at least two consecutive surveys ) (232 days vs. 139 days; P=0.0050; HR 0.704 (0.550-0.905)), as well as the time progression of the fatigue interference (defined as the time to increase of the item fatigue interference on the BFI interference scale by ≥ 1.25 points from the starting value in at least two consecutive surveys) (281 days vs. 139 days; P=0.0008; HR 0.655 (0.510-0.841)) in comparison to the placebo arm.

Due to the increasing significance of the topic in the patient care and lacking data under real life conditions, the transferability of these results from the randomised, controlled clinical Phase III study COU-AA-301 is also to be investigated in this study. For this the questionnaire Brief Fatigue Inventory (BFI) is to be used.

Study objectives

The objective of the NIS planned here is now to ascertain the influence of additional adherence measures on the therapy adherence with Abiraterone with patients with metastatic castration resistant prostate carcinoma in comparison to the application without adherence measures under everyday conditions. Accordingly, the following assessment objectives result:

*Primary:*

Rate of the therapy discontinuations after 3 months for other reasons than disease progression or start of a new carcinoma therapy

*Secondary:*

- Rate of the therapy discontinuations after 6 months for other reasons than disease progression or start of a new carcinoma therapy
- Reasons for, and time until therapy discontinuation
- Adherence of the patient at baseline and subsequently every 3 months, according to questionnaire (i.e. Morisky scale 4-Item-Version MMAS-4)
- ‘Overall Survival‘ over the entire observation period
- Median maximum relative PSA decrease in comparison to baseline (laboratory)
- Change of the FACT-P-Score in month 3 and 6 in comparison to baseline
- Change of the fatigue score (Brief Fatigue Inventory (BFI) in month 3, 6, 12 and month 3 after the end of therapy in comparison to baseline for the patients who are documented after implementation of the Amendment II.
- Safety and tolerability of the therapy (AEs/SAEs; also from questionnaires and the regular controls of the progression)
- Percentage of the patients, who experience pain reduction according to numeric rating-scale (NRS, i.e. BPI Item #3: Decrease of the pain intensity in the last 24 hours by ≥ 30% at 2 consecutive assessments at least 4 weeks apart without increase of the intake of analgesic substances according to WHO grade scheme. Inclusion of patients with entry value ≥ 4 for the statistical analysis)

# 4 Medicinal product information

Abiraterone (marketing name Zytiga®) is an active substance, which in combination with Prednisone or Prednisolone (P/P) is used in adult men for the treatment of metastatic, castration resistant prostate carcinoma (mCRPC), whose disease during or after a chemotherapy containing Docetaxel is progressive, respectively whose disease after the failure of the androgen deprivation therapy has an asymptomatic or mildly symptomatic progression and for whom a chemotherapy is not yet clinically indicated.

Abiraterone has a new type of effect mechanism that is based on the principle of androgen biosynthesis inhibition. Abiraterone acetate is transformed in vivo to Abiraterone and selectively and irreversibly inhibits CYP17, a key enzyme in the androgen biosynthesis in the testicles, adrenal glands, as well as in the prostate tumour tissue. Through this fundamental inhibition of the androgen biosynthesis a deeper reaching androgen deprivation is achieved (serum testosterone level <1 ng/dL) than with conventional deprivation therapy [17-19]. In clinical studies, in spite of existing castration resistance Abiraterone showed a high efficacy with significant improvement in the overall survival of patients with progressive mCRPC under or after chemotherapy with Docetaxel, and with mCRPC patients with an asymptomatic and mildly symptomatic progression of the disease after

failure of the androgen deprivation, for whom a chemotherapy is not yet clinically indicated [5,6].

Comprehensive product information can be reviewed in the current summary of product characteristics [20] of Zytiga®.

# 5 Study design

The study presented here is structured on the basis of a non-interventional design, which is characterised in that the pharmacological therapy including diagnosis and monitoring that is to be investigated will be performed according to the usual medical treatment practice. This means that the physician and patient do not follow a therapy regime that has been previously defined for the study, but the maximally uninfluenced treatment routine and observation of it is to stand in the foreground. The relevant question of the therapy adherence is especially well able to be recorded during the application of the therapy in question for prostate carcinoma under real life conditions, as over the course of this NIS the efficacy and safety of the pharmacological intervention is not to be investigated.

In the NIS presented here, now the benefit of an adherence program under real life conditions is to be investigated under the therapy of prostate carcinoma, i.e. no interventions will be undertaken in the medication therapy (such as e.g. medication changes), as it does not involve gaining understanding in relation to the pharmacological therapy, but gaining understanding on the adherence with the usual application of the medicinal product through the patient. Abiraterone will thereby exclusively be observed and documented during the application within the scope of the marketing authorisation and prescription regulations without special, study-specific medication being used. This is performed according to the guidelines of the federal joint committee on the prescription of medicinal products in statutory health care (medicinal products directive/AM-RL) dated December 18, 2008, in the last amendment dated June 18, 2009, published in the Federal Gazette No. 119, page 2786 with validity since August 14, 2009. The patient assignment to the possible adherence program (study arm A) or not (study arm B) is thereby performed randomly by means of a centre-wise, cluster-randomised distribution [21,22].

# 6 Patient collective and documentation criteria

The identification/selection of patients takes place after the decision about the therapy has been made within the scope of the routine treatment. The patients will be informed about the inclusion in the non-interventional study and be asked to give their consent for possible participation in the planned adherence program. In addition to this, before the beginning of the documentation the written consent of the patient will be acquired for a source data alignment in conformity with the directives of the data protection legislation.

Approximately 780 patients at approximately 100 specialist medical (i.e. urological and oncological) study centres throughout Germany are to be included for the prospective observation and documentation of a standard outpatient therapy with Abiraterone in combination with Prednisone or Prednisolone. Thereby it is intended that 390 patients are to participate in the planned adherence program regarding the compliance (Arm A), and 390 patients are to be treated without special adherence measures (Arm B) under routine conditions.

The study centres are to be equally (i.e., respectively 50) randomly assigned to one or the other arm centre-wise, through which it is ensured that all patients at a centre are always treated in the same way. The random assignment of the centres is achieved through cluster randomisation. Effective strength, pharmaceutical form, and application scheme of Abiraterone are thereby within the scope of the marketing authorisation according to the summary of product characteristics [20] and the consideration of a patient for this NIS only takes place after the decision about the individual therapy has already been taken.

The identification of suitable patients is performed according to the following criteria:

- Men at an age of at least 18 years
- Diagnosis of a metastatic, castration resistant prostate carcinoma,
  1. which is progressive under or after a chemotherapy containing Docetaxel
  or
  2. with asymptomatic or mildly symptomatic progression of the mCRPC after failure of the androgen deprivation therapy, when a chemotherapy is not yet clinically indicated.
- Therapy with Abiraterone-based treatment regime
- Signed patient information and declaration of consent is available at the beginning of the documentation

Study specific exclusion criteria are not defined. The documentation of patients will be excluded, who participate in other clinical studies during the observation period.

The documentation takes place in electronic form with record forms that are made available by JANSSEN-CILAG. To be able to assign the specified patient numbers to the individual patients the participating physician will receive a patient identification form, which remains with his records as confidential document.

# 7 Study parameters and study duration

Diagnostic and therapeutic measures are not specified within the scope of this non-interventional study, they are solely the decision of the treating physician. However, to allow a systematic assessment of the surveyed data the type and scope of the documentation is standardised in an electronic documentation form, as well as the time frame for the observation during the planned study progression.

The following <u>parameters</u>, insofar as surveyed, are to be documented:

- Demographic data (year of birth, gender, height, weight)
- Anamnestic data (age on initial disease manifestation, diagnosis, relevant accompanying diseases, accompanying medication)
- TNM stage (according to AJCC tumour, nodes, metastasis system, 7. edition) of the prostate carcinoma at the time of the beginning of therapy
- Gleason-Score of the prostate carcinoma (time of the surveying variable) Sociodemographic data regarding social contacts and education status
- Type, dosage and duration of the previous carcinoma therapy including reason for the current adjustment, respectively change
- Dosage of Abiraterone and relevant accompanying medication (such as e.g. Prednisone/Prednisolone) as of beginning of study/treatment
- Questions and measures on adherence

- Change and assessment of the clinical symptoms over the progression through the investigator (surveying of specific parameters, but also application of instruments such as questionnaires for general assessment)
- Adverse events (AE)
- Concluding assessment of the efficacy and tolerability of Abiraterone through the treating physician

The surveying of the laboratory parameters and results from imaging procedures such as CT or MRT will be exclusively documented according to the routine diagnostics that is usual at the respective centre. Regarding the laboratory tests the regular documentation of the following parameters is to be aspired to the greatest extent possible:

- PSA, ALT, AST, INR, AP, LDH, bilirubin, creatinine, potassium, small blood picture

Regarding the planned evaluation of the adherence within the scope of the therapy under everyday conditions, the attempt is to be made to achieve the most complete processing of the following questionnaires:

- Charlson-Score (Co-Morbidity [23]); FACT-P (quality-of-life [21]); NRS/ (pain, only question 3 from Brief Pain Inventory[21]); Morisky-scale 4-Item-Version MMAS-4 (adherence, [24]), Brief Fatigue Inventory (BFI) [31]), as well as the following <u>additional questions</u>:
  - Do you drink alcohol?
  - What additional support would you wish from your doctor?

At the beginning of the study the patient should answer the following socioeconomic questions:

- o Do you live together with one or several individuals in one household?
- o Do you receive support or help through another person regarding the regular intake of your medicines?
- o What is your family status?
  A: I am married and live together with my spouse
  B: I am married and live separately from my spouse
  C: I am single
  D: I am divorced
  E: I am a widower
  F: Different family status, namely _____________
- o What professional qualification do you have?
  A: I am still under professional training (trainee, student)
  B: I do not have any professional qualification and am not under professional training
  C: I have completed a professional company vocational training (apprenticeship)
  D: I have completed a professional school training (technical college, commercial college)
  E: I have completed training at a technical college, master school or engineering school, respectively professional or specialist academy
  F: I have a polytechnic degree
  G: I have a university degree
  H: I have a different professional qualification, namely:

Study duration

The study is planned to start in QIII 2013 and end in QIII 2018. The patients can be included up until approximately QIII 2016. The study progression and the documentation duration extend over the entry visit on day 1 (Baseline) as well as controls of the progression every 2 weeks in the first 3 months and then every 3 months. If a patient discontinues with the Abiraterone therapy during the documentation period, then the survival (OS) is recorded for a period of maximally 2 years, i.e. 2 years as of the beginning of the documentation (by means of visit or by telephone). The documentation ends for the individual patient at the latest after 2 years.

The adherence measures for study arm A are defined in the following:

- educative video for the patient on the therapy with Abiraterone (mode of action, correct application, possible side-effects) at the entry visit.
  During the control of progression in week 2 the patient will be asked:
  *"How often have you seen the training video on Zytiga®?"*
- telephone reminder service through the study centre during the first 12 weeks alternating 2-weekly with the 2-weekly visits (i.e. weekly contact in person or by telephone). Subsequently the further follow-up is performed by monthly telephone calls alternating with possible monthly visits, which are documented every 3 months (i.e. if possible 2-weekly personal contact or by telephone). Within the scope of the telephone calls and visits the adherence and adverse events are surveyed under the treatment using a structured interview guide, so that help can be offered at an early time, respectively countermeasures can be initiated with adverse events. This guide includes the following questions:

Question 1: How are you today?

- o Have any complaints occurred since the last contact?

Question 2: Did you have any problems with the intake of Zytiga® or Prednisone?

- o Did you have any problems in swallowing the tablets?
- o Did you have any difficulties taking the tablets on an empty stomach?
- o Do you have any problems with the regularity of the medication intake?
- o Have you forgotten to take Zytiga® or Prednisone since our last contact?

- As of the beginning of the study the patient can document the daily intake of Zytiga® and Prednisolone in a diary.
- A special dosage card can facilitate the planning of the intake of the medication for the patient.

# 8 Flowchart

The following compilation summarises the data to be documented over the course of the study in the form of a flowchart. The object of the documentation and the corresponding times are suggestions that have been derived from the routine course of an Abiraterone therapy, and which are only to be recorded for this NIS if possible. Study-specific clinical or invasive measures are not provided for due to the non-interventional character of the study.

<u>Diagram of the documentation times</u>

| **Documentation type** | **Baseline** | **Controls of progression** | | **Follow-up observation phase** |
|---|---|---|---|---|
| **Time** | **Week 1; Day 1** | **every 2 W up to W 10** | **every 3 M from W 12 until T/D end** | **every 3 M after T-end until D-end** |
| Patient consent | X | | | |
| Anamnesis with sociodemographic, socioeconomic data, accompanying diseases according to Charlson-Score | X | | | |
| <u>Arm A:</u> Training video on the therapy and telephone reminder service **1)**; optional: Issue of patient diary and dosage card | X | X **1)** | X **1)** | |
| Imaging procedures | X | | X | |
| Blood pressure, weight | X | X | X | |
| PSA value | X | X | X | |
| Safety laboratory | X | X | X | |
| Abiraterone therapy | X | X | X | |
| AE/SAE documentation with OS | | X | X | |
| ADR/SADR documentation with OS | | | | X **3)** |
| Accompanying medication | X | X | X | |
| <u>Questionnaires that are completed by patients:</u> | | | | |
| FACT-P-Score (quality-of-life) | X | | X **2)** | |
| Fatigue questionnaire Brief Fatigue Inventory**5)** | X | | X | X **4)** |
| NRS (pain scale) | X | | X | |
| Morisky-scale/ MMAS-4 (adherence measurement) | X | | X | |
| <u>Arm A</u>: Video training questionnaire | | only in W 2 | | |

W: Week; M: Month; OS: Overall Survival; T/D-End: Therapy or documentation end

**1)** **Only Arm A**: **Telephone reminder service** through the study centre 2-weekly in the first 12 weeks alternating with the 2-weekly visits (i.e. weekly contact in person or by telephone).
Subsequently 4-weekly calls alternating with possibly one visit per month, which are documented every 3 months
(i.e. insofar as possible 2-weekly contact in person or by telephone).

**2)** Only in month 3 and 6

**3)** Documentation end is maximally 2 years after the beginning of the documentation

**4) 3 months after end of therapy**

**5) Only for patients, who are documented after implementation of Amendment II**

## 9 General information on the drug safety

A prompt, exact and complete reporting and verification of the information on drug safety from clinical studies is decisive for the protection of the patients, the study physicians and the sponsors of such studies, and is specifically required by the marketing authorisation authorities throughout the world. All clinical studies that are conducted by JANSSEN-CILAG or companies appointed by JANSSEN-CILAG are organised worldwide according to specified procedures and according to monitoring authority requirements, so as to ensure correct reporting of information on drug safety.
Within the scope of the study, depending on the study phase (e.g. no medication under follow-up) adverse events (AEs/SAEs), respectively treatment associated side-effects as well as product complaints and newly occurred pregnancies of the partners are recorded. The necessary timely notification to JANSSEN-CILAG is described under point 8.2. Every employee from the study personnel receives a training course on drug safety.

JANSSEN-CILAG undertakes the notification obligations towards the authorities according to the current legislative situation and prepares a concluding report with all adverse events that are documented in the CRF.

### 9.1 Definitions

Adverse event (AE)

An adverse event is every unfavourable medical event that occurs with a patient after administration of a medicinal product (regardless of whether it is an investigational drug or not), independent of whether a causal relationship is assumed with the treatment. This can be every undesirable, unintentional clinical sign (including a deviating laboratory value or the lack of an expected pharmacological effect), symptom or disease, which stands in a temporal relationship with the administration of a medicinal product (investigational product or not) (definition based on the international conference on harmonisation [ICH]).
This includes every event that newly occurs or becomes worse in severity compared to the starting condition, or any deviating results of any diagnostic procedures that are performed during the routine clinical practice.

Side effect / Adverse drug reaction (ADR)

An adverse drug reaction is defined as a harmful and unintentional reaction towards a medicinal product (trial preparation not). The term "Reaction towards a medicinal product" means that a causal relationship between a medicinal product and an adverse event is possible, probable or very probable.
In contrast to an adverse event, an ADR is characterised through the fact that a causal relationship is assumed between the medicinal product and the event. All adverse events with which the reporting physician or the sponsor assume there to be a plausible causal relationship with a medicinal product are regarded as ADRs.

Serious adverse event (SAE) or serious adverse drug reaction

According to the ICH and EC guidelines on pharmacovigilance for medicinal products for use in humans a serious adverse event [OR a serious adverse drug reaction] is any unfavourable medical event [OR any adverse drug reaction], which independent from the dosage:

- leads to death
- is life-threatening, (i.e. at the time of the occurrence of the event, the patient was in a life-threatening situation; this does not mean an event that may have theoretically led to death if it would have been more serious)
- requires a hospital stay or an extension of it
- leads to a remaining or significant disability / inability to work
- leads to a congenital deformity/ congenital defect as a consequence
- is an assumed transmission of any infectious agent through a medicinal product
- is medically relevant*

  *It should be decided according to medical and scientific discretion, whether further situations are to be considered as serious, e.g. events that are not immediately life-threatening, or lead to death, respectively hospital stay, but represent a significant endangerment or require a medical intervention to prevent it coming to any of the above-mentioned consequences. For reports about hospital stays the serious symptom, respectively the diagnosis that led to hospitalisation must be documented and listed as SAE.

  Hospital stays for the following reasons are not required to be reported as serious events:

  - Medicinal product application
  - Social indication
  - Surgery or other measures that were planned and documented before inclusion in this study
  - Inpatient curative stay

Disease progression should not be documented as designation for a (serious) adverse event; instead of this signs and symptoms of the clinical consequences that result from the disease progression are reported.

Not listed (unexpected) adverse event

An adverse event is regarded as "not listed" when with relation to the type and severity it is not consistent with the corresponding reference information on drug safety. The degree of anticipation of an adverse event depends on whether it is listed in the corresponding reference information on drug safety (e.g. summary of product characteristics)

NOTE: The "not listed" status of an event is only relevant for the reporting obligations of the sponsor. It does not determine the notification requirements of the participating physician towards the sponsor or the marketing authorisation holder.

Product complaints with/without AE/SAE

A complaint on the product quality (product quality complaint - PQC) is defined as any suspected product deficiency in relation to the manufacturing, labelling or packaging, i.e. any dissatisfaction in relation to the identity, quality, shelf life or reliability of a product, including the intactness of its labelling or packaging. A PQC can have effects on the safety and efficacy of the product. A prompt, exact and complete notification and analysis of the PQC information from studies is decisive for the protection of the patients, for the physicians and the sponsor, and is specifically required by the marketing authorisation authorities worldwide. The

sponsor has specified procedures according to the monitoring authority requirements worldwide to ensure correct reporting of PCQ information; all studies that are performed by the sponsor, or through companies associated with the sponsor are performed according to these procedures.

Combined product complaints are associated with an AE/SAE, respectively suspected product deficiencies can have an AE/SAE as consequence. If the deficiency with a Janssen product is associated with a serious adverse event, then the personnel at the study centre must report the PQC to JANSSEN-CILAG, whereby the timeframe for the notification of a serious adverse event must be observed. At the request of the sponsor, a sample of the concerned product must be stored for further investigation.

**Special notification situations**

Events of interest related to the safety with a product from JANSSEN, which require a notification and/or a safety assessment are amongst others, but not restricted to:

- (Maternal and paternal) medicinal product exposition during the pregnancy (see section 9.3)
- Overdose of a product from Janssen
- Assumed misuse/abusive use of a product from Janssen
- Unintentional or random exposition towards a product from Janssen
- Any absence of the expected pharmacological effect (e.g. lacking effect) of a product from Janssen
- Medication errors with a medicinal product from Janssen (with or without exposition of the patient towards the product from Janssen, e.g. mix up of medicinal product names)
- Assumed transmission of any infectious agent through a medicinal product
- Unexpected therapeutic or clinical benefit through the application of a product from Janssen

These safety events may possibly not fulfil the definition of an adverse event; from the strategic perspective, however, they are treated in the same way as adverse events. Special situations should be documented on the page for adverse events of the CRF. Any special situation, which fulfils the criteria of a serious adverse event, should be documented on a form for serious adverse events and reported to JANSSEN within 24 hours after it becomes known.

Causality assessment

For every AE an assessment of the causal relationship between the adverse event and the medication under observation should be undertaken according to the following definition:

*No causal relatedness (not related)*

An event, which (according to the assessment of the treating physician) does not stand in causal relationship with the use of the medication under observation.

*Improbable (*doubtful*)*

An event for which a different cause is more probable, e.g. an accompanying medication/concomitant disease, or the time of the occurrence of the event makes a causal relationship with the medication under observation appear to be unlikely.

*Possible*

An event that may possibly have been caused through the application of the medication under observation. A different explanation, e.g. accompanying medication/concomitant disease is not conclusive. The temporal relatedness is appropriate; therefore a causal relatedness cannot be excluded.

*Probable*

An event that may possibly have been caused through the application of the medication under observation. The temporal relatedness indicates a causal relationship (e.g. confirmed through discontinuation test). An alternative explanation (e.g. accompanying medication/concomitant disease) is less probable.

*Very likely*

An event that is listed as possible adverse reaction and is not able to be conclusively explained by other means (e.g. accompanying medication/ concomitant disease). The temporal relationship with the application of the medication highly suggests itself (e.g. confirmed through discontinuation test and re-exposition).

## 9.2 Documentation and notification of adverse events

The beginning of the documentation of adverse events is as a general principle performed as of the first medication intake, up until the beginning of the follow-up observation phase. The documentation of pregnancies begins with the first medication intake (this means pregnancies that occur with the partners of the male study participants).

All AEs/SAEs (including cases of death) are recorded by means of the electronic CRFs. Every pregnancy must be reported on the form sheet "Pregnancy Notification Form". Every product complaint on Janssen products must be formlessly forwarded to JANSSEN-CILAG.

<u>Deadlines</u>

Every SAE as well as pregnancy notifications and product complaints must be reported to JANSSEN-CILAG within 24 hours, after they have become known to the physician.

This is performed by means of entry in the electronic CRF and printout, signature of the form through the treating physician and sending of the form by Fax to JANSSEN-CILAG (**Fax: 02137-952-357**). In the event that an electronic connection is not possible, SAE forms are available to the physician in paper form in the provided study file for notification by means of Fax.

<u>Not systematically surveyed adverse events (spontaneous adverse events)</u>

With adverse events and special situations that are not systematically surveyed (e.g. with a different medicinal product than the one(s) under investigation and if the participating physician considers it to be possible that there is a possible, probable, or very likely relationship with a medicinal product (i.e. spontaneous ADR)), then the participating physician must inform the manufacturer of the medicinal product or the respectively responsible authority/monitoring authority as fast as possible through the national spontaneous reporting system.

With ADRs and special situations associated with a not investigated Janssen product the event must be directly reported to JANSSEN-CILAG.
Available reports about spontaneous ADRs will be summarised in the clinical study report.

### 9.3 Documentation and reporting of adverse reactions during the follow-up observation phase

In the follow-up observation phase exclusively adverse reactions will be documented. Serious adverse reactions, as well as newly occurred pregnancies with the partners of the male study participants and product complaints are documented and reported to JANSSEN-CILAG as described under point 8.2 (deadlines).

Documentation/notification of cases of death during the follow-up observation phase
All cases of death during the follow-up observation phase are documented under specification of the causality classification towards the medication under observation and the cause of death. In addition to this, a timely notification to JANSSEN-CILAG is performed, if the treating physician deems there to be an at least possible relationship between the observed medication and the cause of death.

JANSSEN-CILAG GmbH
Dept. Clinical Research
Johnson & Johnson Platz 1
41470 Neuss
Phone: 02137-955-489
Fax : 02137-952-357

Insofar as further information is necessary to clarify the notified reports, JANSSEN-CILAG will contact the physician, respectively the contracted research institute. If JANSSEN-CILAG receives a report about a serious adverse reaction or about a pregnancy for a medication under observation, for which the marketing authorisation holder is not Johnson & Johnson, JANSSEN-CILAG will inform the corresponding marketing authorisation holder.

### 9.4 Other documentation requirements

All additionally received information is to be reported within the scope of a follow-up report to JANSSEN-CILAG according to the above-mentioned procedures/deadlines.

## 10 Supervision/procedure

The supervision and the monitoring of this NIS will be performed through authorised employees of JANSSEN-CILAG GmbH, and/or through employees of a contract research institute assigned by JANSSEN-CILAG. The employees are available to the physician for all questions regarding the execution of the study, as well as the procedure for documentation and notification of adverse events. At the beginning of the study the study plan is issued and discussed in detail. Prerequisite for the beginning is the presence of a signed contract.

# 11 Quality assurance

## 11.1 Documentation

To secure the quality of the data surveyed in this NIS a validation of this data is necessary. The validation is performed through the harmonisation of the documentation in the medical record with the entries in the documentation form (see point 13. Data protection). Prerequisite for this is the careful documentation of all diagnostic and therapeutic measures and findings, as well as all occurred adverse events (AEs and SAEs) in the medical record.

## 11.2 Measures on quality assurance, monitoring

The NIS presented here will be performed on the basis of the ‘Recommendation on the planning, execution and assessment of application observations’ published by the BfArM on November 12, 1998, the draft of the joint recommendation of the BfArM and PEI dated May 09, 2007, and the ‘VFA recommendation for the improvement of the quality and transparency of non-interventional studies (2007)’.

Before the beginning of the documentation the participating physicians will be informed by employees of JANSSEN-CILAG, respectively by a contract research institute authorised through JANSSEN-CILAG about the handling of the electronic data recording, the procedure of the NIS, as well as especially about the handling of adverse events. For the answering of technical questions a hotline at the company acromion GmbH is available under the following telephone number during normal business hours:

+49 / 2234 / 20373737

Authorised employees of JANSSEN-CILAG subject to confidentiality or appointees of the sponsor will verify the authenticity and the quality of the documented data over the course of the NIS. The participating physician grants the employees the verification of the study progression as often as is necessary. During all these visits he provides the access to the electronic documentation system, and is available for enquiries.

The employee randomly verifies the data (patient-specific and disease relevant parameters, target parameters, results of tests and available laboratory values) for completeness and plausibility according to the monitoring directives. This takes place under observance of the data protection regulations through direct review of the medical record (direct source data harmonisation).
A further verification of the data for completeness and plausibility will be performed by the appointed statistical institute. The participating physician undertakes the obligation to answer questions resulting from this.

The written consent with which the patient is informed about the participation in the NIS and the forwarding of the data and in which he/she agrees to a direct review of his/her medical records through employees of JANSSEN CILAG or authorised personnel under consideration of the data protection laws, is prerequisite for documentation of the treatment within the scope of this investigation.

## 11.3 Audit

Due to quality assurance reasons, JANSSEN-CILAG reserves the right to conduct an audit, respectively have it performed by appointed third parties. The participating physician undertakes the obligation to cooperate accordingly in this.

# 12 Statistics

## 12.1 Random sample calculation

The case number calculation is based on the data of the marketing authorisation study with Abiraterone in the post-chemo-setting, as at the time of the study design the authorised indication for Abiraterone was solely in the post-chemo-setting. In the so-called Cougar-AA-301 study (Post-Chemo-Setting) 18% of the patients prematurely discontinued the treatment with Abiraterone within 12 weeks after the start of the therapy, and a further 21% after 24 weeks [21]. The proportion of the patients who have begun a first, second or subsequent cycle was largely equally distributed on consideration of the first 8 treatment cycles, so that the rate of the treatment discontinuations appears to rather follow a continuous progression. In total 34% of the patients discontinued the treatment with Abiraterone for other reasons than disease progression or due to the beginning of a new or different carcinoma therapy. It is assumed that the required number of patients with discontinuation of an Abiraterone treatment for other reasons than disease progression or new carcinoma therapy outside of a clinical study is higher due to poor adherence. The difference of 10% to month 3 in treatment losses between the two patient groups is thereby regarded as being clinically relevant, corresponding to a ‘Number-needed-to-treat‘ of approximately 10. On the assumption of the best possible discontinuation of 18% with month 3 the expected effect-efficacy gap between the data from the Cougar-AA-301 study and the everyday conditions would be able to be completely closed [21]. In association with this a difference in the discontinuation rates of 10% in week 12 (i.e. 18% vs. 28%) would be identified with 80% power on the basis of a sample size of 594 assessable patients (297 per treatment arm), which represents an acceptable assumption for the test for superiority with a 5% significance level. As a loss for the efficacy sample must be taken into consideration due to the alternating treatment regime between the centres (multiplication of the case number by $1+[m-1]\rho$), a total of 701 patients are to be included (estimated cluster correlation coefficient: $\rho=0.02$; mean cluster size: $m=10$); [22]. To be able to allow a total dropout rate of approximately 10% at the beginning of the study, therefore a total of at least 780 patients (390 per arm) must be recruited.

Through the opening of the study according to the extended indication of Zytiga (additionally pre-chemo setting) per Amendment I, due to the planned explorative recalculation (see section 12.2), which was already planned subsequent to the first interim analysis after a total of 200 patients, the case number at the time of Amendment I was not changed. In addition, at this time it is unclear which assumptions about the expected proportion values of pre and post chemo patients will be able to be made within this study, so as to be able to make an assumption for the dropout rate of the totality of the patients.
To be able to identify existing differences in the totality of the patients with sufficient selectivity, therefore a conservative approach was selected, and the higher discontinuation rates of the post-chemo patients (i.e. 18% vs. 28%) were confirmed for the planning of the case number.

For the marketing authorisation study in the pre-chemo setting (Cougar-AA-302 study) the following figures are available: In the Cougar-AA-302 study 7% of the patients prematurely discontinued the treatment with Abiraterone within 12 weeks after the start of the therapy, and a further 12% after 24 weeks. The proportion of the patients who have begun a first, second or subsequent cycle was largely equally distributed on consideration of the first 8 treatment cycles, so that the rate of the treatment discontinuations appears to rather follow a continuous

progression. In total 17% of the patients discontinued the treatment with Abiraterone for other reasons than disease progression or due to the beginning of a new or different carcinoma therapy. It is assumed that the required number of patients with discontinuation of an Abiraterone treatment for other reasons than disease progression or new carcinoma therapy outside of a clinical study is higher due to poor adherence.

interim analysis

Within the scope of the first interim analysis from March 2015 with a total of 203 patients, for the primary endpoint "Rate of therapy discontinuations after 3 months for other reasons than disease progression or start of a new therapy" no difference was shown in the two observation groups (6.0% (6 /100 patients) in adherence arm vs. 3.9% (4/103 patients) in the non-adherence arm. The total rate of therapy discontinuations after 3 months was 19.0% (19/100 patients) vs. 12.6% (13/103 patients). The analysis of the patient distribution regarding pre and post chemo segment showed a clear imbalance in favour of a pre-chemo distribution (73.4% (149/203 patients) vs. 26.6% (54/203 patients). The assumptions used for the statistical justification of the case number were not able to be confirmed. At the time of the first interim analysis it remains unclear whether the results of the interim analysis are purely random or are due to unequally distributed influence factors in the two study arms or at individual study centres. Since in the adherence arm only two more patients terminated treatment than in the non-adherence arm and both events according to different reasons ( “other reasons” or “intolerance of therapy”) we consider the results to be randomly explained rather than caused by an inbalance of influencing factors in both arms.

To verify whether the discontinuation rates per study arm and for the subgroups of the pre and post chemo patients observed during the first interim analysis are able to be confirmed over the further duration of the study, it was therefore decided to continue with the study.

## 12.2 Statistical analysis

The statistical assessment and tabular compilation of the results will be performed through an independent statistical institute. The exact details on the assessment, including the definition of comparisons between the study arms will be specified in a separate statistical analysis plan (SAP) after the end of the documentation and before the beginning of the assessment. All calculations as well as the compilation of the statistical tables will be performed with the statistical program package SAS®.

In the assessment of the efficacy and tolerability all patients will be included who were included in this non-interventional study (‘intent-to-treat‘ analysis). The demographic and clinical variables at the beginning of the study will be descriptively summarised. Study plan deviations will be listed and counted out. The statistical analysis will take place with methods of descriptive and explorative statistics.

After 100 patients per arm (i.e. 200 patients in total), who were documented for 3 months, a first interim assessment is to be performed. Based on the rate of the therapy discontinuations (in total per study arm and in the subgroup of the pre chemo and post chemo patients) after 3 months for other reasons than disease progression or beginning of a new carcinoma therapy, an explorative recalculation of the case number and a clinical assessment will be performed under consideration of the observed therapy adherence.

Should the results of the interim assessment including relevant clinical aspects, as well as the recalculation of the case number not allow the expected benefit of the adherence program to be identified, then JANSSEN-CILAG reserves the right to discontinue with the study. If over the study progression further interim assessments are considered to be expedient, then these would be performed at later times. A respectively detailed description, as well as corresponding definitions on the design of an interim assessment will then be adopted in the SAP.

All adverse events will be coded and listed according to MedDRA. Type and frequency of the adverse events will be represented in table form according to the MedDRA terminology (i.e. primary System Organ Class and Preferred Term) and grouped and analysed under various aspects, e.g. causal relationship. Especially considered will be patients who have discontinued the study due to an adverse event with whom a serious adverse event occurred.

The assessment and interpretation of the data of the fatigue questionnaire on the quality-of-life takes place according to the assessment guidelines in the subgroup of the patients, who were included in the study after the coming into force of Amendment II.

All documented patient data will be represented parameter-wise in individual value lists, and depending on the scale level summarised in table form with descriptive statistical parameters, i.e. with continuously variable arithmetic mean value, standard deviation, median, minimum, maximum, 1. and 3. quartile, respectively with discrete variables specification of the frequency distribution (number %) of the patients with percentage (if not mentioned differently) in relation to the total number of the patients. Where it appears to be expedient, the numeric results will be supplemented through corresponding graphic representations.

<u>Update after first interim analysis 2015</u>

The first interim analysis was completed in March 2015. Thereafter it has been decided to continue with the study to investigate if the withdrawal rates observed per study arm and for the subgroups in the pre- and post-chemo setting can be confirmed during the further progression of the study. Furthermore a second interim analyses will be conducted at month 6 including the analyses of adherence parameters and possible influencing factors on adherence in both groups.

# 13 Ethics Committee

JANSSEN-CILAG will have the study and all amendments evaluated by an independent ethics committee. The following will be submitted:

- Study plan
- Documentation form (paper printout of e-CRF; if applicable as draft version)
- Patient information form and declaration of consent
- Adherence program
- Curriculum Vitae of the principal investigator
- Other documents (e.g. patient diaries and questionnaires)

# 14 Data protection

The personal data acquired within the scope of the study after the declaration of consent of the study participant, especially findings, are subject to medical confidentiality and the data protection law regulations. It will be recorded and

pseudonymised at the company Acromion GmbH, Frechen and stored for the duration of 10 years. During the pseudonymisation the name and other identification characteristics will be replaced through a code to exclude the identification of the study participant or to make it significantly more difficult.

Apart from the treating physician (head of the study) only his deputy has access to the code. The personal data will be made available for review through the employees of the sponsor or their appointees for verification of the correct execution of the study. All of these individuals that may possibly review the patient record are subject to confidentiality.

The assessment and use of the data takes place in pseudonym form. A forwarding of the surveyed data within the scope of the study only takes place in anonymised form. The same applies for the publication of the study results.

The study participants have the right to request information from their treating physician about the personal data recorded about them.

This study has been subject to consultation through the responsible ethics committee. The responsible state authority can, if applicable, be provided access for review of the study documents.

As soon as the research purpose permits, the code will be deleted and the recorded data will thereby be anonymised.

In the event of a revocation of the declaration of consent the already recorded data will also be deleted or anonymised and used further in this form. A revocation of already anonymised data is not possible.

# 15 Results/publication

All study data and results, including AEs/SAEs will be recorded, documented, stored and forwarded by means of eCRF. As is required by law the corresponding source data remains at the study centre, and can be reviewed there, respectively is also available there. The overall results of the NIS are to be represented in an integrated concluding statistical and medical report, including all safety relevant data.

A possible publication of the overall results takes place under the coordination of JANSSEN-CILAG. Partial results cannot be published without consultation with JANSSEN-CILAG, as well as the approval of the head of the study.

In accordance with the "VFA recommendations on the improvement of the quality and transparency of non-interventional studies" (2007), before the beginning of the study JANSSEN-CILAG will make the summary of the study plan publicly available.

# 16 Administrative issues/notification obligation

This non-interventional study will be performed on the basis of the ‘Recommendation on the planning, execution and assessment of application observations’ published by the BfArM on November 12, 1998, the draft of the joint recommendation of the BfArM and PEI dated May 09, 2007 and the ‘VFA recommendation for the improvement of the quality and transparency of non-interventional studies’ (2007), as well as the ‘Notice to Marketing Authorisation Holders – Pharmacovigilance Guidelines’ of the EMEA (European Agency for the Evaluation of Medicinal Products).

JANSSEN-CILAG, respectively the contract research institute authorised by JANSSEN-CILAG, will notify the study and all amendments according to §67,

section 6, German pharmaceuticals act to the federal Institute for drugs and medical devices, as well as the National Association of Statutory Health Insurance Physicians and the leading Associations of the health insurance providers (if applicable represented through the BKK National Association) and undertake the regulatory responsibilities according to the applicable German pharmaceuticals act (§ 63).

The study will be additionally posted on the web site of the VFA.

# 17 Archiving

All recorded data as well as documents are the property of the sponsor. All documents will be archived for at least 10 years for later access and assessment.

# 18 Responsibilities

<u>Head of study</u>

PD Dr. med. Henrik Suttmann
Urology Hamburg
Harksheider Str. 22
22399 Hamburg

<u>Study coordination and enquiries</u>

Dr. H.-W. Grosch
Global Clinical Operations
Janssen-Cilag GmbH
Johnson & Johnson Platz 1
41470 Neuss

<u>Medical and scientific concept</u>

Dr. med. Natasha Schuier
Janssen-Cilag GmbH
Address see above

IFOM - Institut für Forschung in der Operativen Medizin (Institute for research in operative medicine)
Chair for surgical research
Faculty for health, Department for human medicine

Univ. Prof. Dr. Prof. h.c. Edmund A. M. Neugebauer
Mrs. Dr. M. Eikermann, Mr M.D.H. D. Pieper, Mr Dipl. Ges. Ök. T. Mathes
Private university Witten/Herdecke gGmbh
Ostmerheimer Str. 200, Haus 38
51109 Cologne

<u>Pharmacovigilance</u>

Dr. H. Euwens
Janssen-Cilag GmbH
Address see above

<u>Monitoring</u>

Quintiles Commercial Germany GmbH
Schildkrötstraße 17-19
D-68199 Mannheim

<u>Biometry</u>

Dipl.-Stat. W. Baurecht
Acromion GmbH
Europaallee 27-29
50226 Frechen

# 19 Literature list


1. Empfehlungen des BfArM und des PEI zur Planung, Durchführung und Auswertung von Anwendungsbeobachtungen in der aktuellen Version vom 07. Juli 2010
2. Notice to marketing authorization holders – Pharmacovigilance guidelines. *cpmp/phvwo/108/99 corr. 28.01.1999*
3. Vfa-Empfehlung zur Verbesserung der Qualität und Transparenz von nicht-interventionellen Studien (2007)
4. Janssen-Cilag: Der Nutzen von Abirateronacetat. Auszüge aus den Inhalten des beim G-BA eingereichten Dossiers zur frühen Nutzenbewertung von Zytiga®. Oktober 2011
5. De Bono JS, Logothetis CJ, Molina A, Fizazi K, North S, Chu L, et al. Abiraterone and increased survival in metastatic prostate cancer. *N Engl J Med 2011; 364(21): 1995-2005.*
6. Rathkopf et al. ASCO GU 2013; Abstract 5 (Oral Presentation)
7. Heck MM,·Höppner M, Horn T, Thalgott M, Gschwend JE, Retz M et al. Abirateron- und Cabazitaxel-Härtefallprogramm. Erste klinische Erfahrung bei Docetaxel-vorbehandelten, kastrationsresistenten Prostatakarzinompatienten. *Der Urologe 2012: 1-8*
8. Sabatè E. Adherence to long-term therapies. Evidence of action, Geneva, *World Health Organization (Hrsg.) 2003: 1-194*
9. Foster JM, Smith L, Bosnic-Anticevich SZ, Usherwood T, Sawyer SM, Rand CS, Reddel HK. Identifying patient-specific beliefs and behaviours for conversations about adherence in asthma. *Intern Med J 2012, 42(6): e136-e144*
10. Wood L. A review on adherence management in patients on oral cancer therapies. *Eur J Oncol Nurs 2011 Nov 1*
11. Halfdanarson TR, Jatoi A. Oral cancer chemotherapy: the critical interplay between patient education and patient safety. *Curr Oncol Rep 2010, 12(4): 247-252*
12. Foulon V, Schöffski P, Wolter P. Patient adherence to oral anticancer drugs: an emerging issue in modern oncology. *Acta Clin Belg 2011, 66(2): 85-96*
13. Hershman DL, Shao T, Kushi LH, Buono D, Tsai WY, Kwan M et al. Early discontinuation and non-adherence to adjuvant hormonal therapy are associated with increased mortality in women with breast cancer. *Breast Cancer Res Treat. 2011; 126(2): 529-537*
14. Noens L, van Lierde MA, De Bock R, Verhoef G, Zache´e P, Berneman Z et al. Prevalence, determinants, and outcomes of non-adherence to imatinib therapy in patients with chronic myeloid leukemia: theADAGIO study. *Blood 2009; 113(22); 5401-5411*
15. Ruddy K, Mayer E, Partridge A. Patient adherence and persistence with oral anticancer treatment. CA *Cancer J Clin 2009, 59(1): 56-66*
16. Eichler HG, Abadie E, Breckenridge A, Flamion B, Gustafsson LL, Leufkens H et al. Bridging the efficacy-effectiveness gap: a regulator's perspective on addressing variability of drug response. *Nat Rev Drug Discov. 2011; 10(7): 495-506*
17. Attar RM, Takimoto CH, Gottardis MM. Castration-resistant prostate cancer: Locking up the molecular escaperoutes. *Clinical Cancer Research 2009; 15(10): 3251-3255.*
18. Montgomery RB, Mostaghel EA, Vessella R, Hess DL, Kalhorn TF, Higano CS, et al. Maintenance of intratumoral androgens in metastatic prostate cancer: a mechanism for castrationresistant tumor growth. *Cancer Res 2008; 68(11): 4447-4454.*
19. Locke JA, Guns ES, Lubik AA, Adomat HH, Hendy SC, Wood CA, et al. Androgen levels increase by intratumoral de novo steroidogenesis during progression of castration-resistant prostate cancer. *Cancer Res 2008; 68(15): 6407-6415.*
20. Janssen-Cilag: Fachinformation Zytiga® 250mg Tabletten (aktuelle Version verfügbar auf der Homepage des eCRF)

21. Janssen Research & Development. Statistical Report of Updated Data from Study COU-AA-301 Protocol COU-AA-301; Phase 3 JNJ-212082 (Abirateronee acetate). *EDMS-ERI-23542052: 1.0. June 2011*

22. Killip S, Mahfoud Z, Pearce K. What is an intracluster correlation coefficient? Crucial concepts for primary care researchers. *Annals of Family Medicine 2004; 2(3): 204-208*

23. Charlson ME, Pompei P, Ales KL, et al. A new method of classifying prognostic comorbidity in longitudinal studies: Development and validation. *J Chron Dis. 1987; 40: 373-383*

*24.* Morisky DE, Green LW, Levine DM. Concurrent and predictive validity of a self-reported measure of medication adherence. *Med Care 1986. 24(1): 67-74*

25. Glaus A., Crow R, Hammond S. A qualitative study to explore the concept of fatigue/tiredness in cancer patients and in healthy individuals. *Eur J Cancer Care (Engl). 1996 Jun;5(2 Suppl):8-23.*

26. Hofman M, Ryan JL, Figueroa-Moseley CD, Jean-Pierre P, Morrowa GR. Cancer-Related Fatigue: The Scale of the Problem. *The Oncologist 2007;12(suppl 1):4–10*

*27.* Curt GA, Breitbart W, Cella D, Groopman JE, Horning SJ, Itri LM, Johnson DH, Miaskowski C, Scherr, SL, Portenoy RK, Vogelzang NJ. Impact of Cancer-Related Fatigue on the Lives of Patients: New Findings From the Fatigue Coalition. *The Oncologist 2000;5:353-360*

28. Stone P, Richardson A, Ream E, Smith AG, Kerr DJ, Kearney N. Cancer-related fatigue: Inevitable, unimportant and untreatable? Results of a multi-centre patient survey on behalf of the Cancer Fatigue Forum*. Annuals of Oncology 11: 971-975, 2000*

*29.* Vogelzang NJ, Breitbart W, Cella D, Curt GA, Groopman JE, Horning SJ, Itri LM, Johnson DH, Scherr SL, Portenoy RK. Patient, caregiver, and oncologist perceptions of cancer-related fatigue: results of a tripart assessment survey. The Fatigue Coalition. *Semin Hematol. 1997 Jul;34(3 Suppl 2):4-12.*

*30.* Sternberg CN, Molina A, North S, Mainwaring P,Fizazi K, Hao Y, Rothman M, Gagnon DD, Kheoh T, Haqq CM, Cleeland C, de Bono JS, Scher HI. Effect of abiraterone acetate on fatigue in patients with metastatic castration-resistant prostate cancer after docetaxel chemotherapy. *Annals of Oncology 24: 1017–1025, 2013 doi:10.1093/annonc/mds585*

*31.* Mendoza TR, Wang XS, Cleeland CS et al. The rapid assessment of fatigue severity in cancer patients: use of the Brief Fatigue Inventory. *Cancer 1999;85:1186-1196.*

# 20 Signatures

I have read the observation plan for the non-interventional study with Abiraterone (212082PCR4002) in the final version, including Amendment II, dated July 02, 2015 and hereby declare my consent:

Date: ..........................................................
Head of study
PD Dr. med. Henrik Suttmann

Date: .........................................................
Physician responsible for the study
Dr. med. Natasha Schuier
Janssen-Cilag GmbH

Date: .........................................................
Centre-specific study physician
(Name in printed characters and signature)
